# Recruitment of representative samples for low incidence cancer populations: Do registries deliver?

**DOI:** 10.1186/1471-2288-11-5

**Published:** 2011-01-16

**Authors:** Tara Clinton-McHarg, Mariko Carey, Rob Sanson-Fisher, Elizabeth Tracey

**Affiliations:** 1 Health Behaviour Research Group, Priority Research Centre for Health Behaviour (PRCHB), University of Newcastle, and the Hunter Medical Research Institute (HMRI), Callaghan, New South Wales, Australia; 2 Division of Cancer Information and Registries, Cancer Institute NSW, Eveleigh, New South Wales, Australia

## Abstract

**Background:**

Recruiting large and representative samples of adolescent and young adult (AYA) cancer survivors is important for gaining accurate data regarding the prevalence of unmet needs in this population. This study aimed to describe recruitment rates for AYAs recruited through a cancer registry with particular focus on: active clinician consent protocols, reasons for clinicians not providing consent and the representativeness of the final sample.

**Methods:**

Adolescents and young adults aged 14 to19 years inclusive and listed on the cancer registry from January 1 2002 to December 31 2007 were identified. An active clinician consent protocol was used whereby the registry sent a letter to AYAs primary treating clinicians requesting permission to contact the survivors. The registry then sent survivors who received their clinician's consent a letter seeking permission to forward their contact details to the research team. Consenting AYAs were sent a questionnaire which assessed their unmet needs.

**Results:**

The overall consent rate for AYAs identified as eligible by the registry was 7.8%. Of the 411 potentially eligible survivors identified, just over half (n = 232, 56%) received their clinician's consent to be contacted. Of those 232 AYAs, 65% were unable to be contacted. Only 18 AYAs (7.8%) refused permission for their contact details to be passed on to the research team. Of the 64 young people who agreed to be contacted, 50% (n = 32) completed the questionnaire.

**Conclusions:**

Cancer registries which employ active clinician consent protocols may not be appropriate for recruiting large, representative samples of AYAs diagnosed with cancer. Given that AYA cancer survivors are highly mobile, alternative methods such as treatment centre and clinic based recruitment may need to be considered.

## Background

### Importance of research for improving cancer control

Research plays an important role in the advancement of cancer prevention, screening, treatment and follow-up care [1-3]. It has led to improvements in morbidity and mortality for people with cancer over the last 20 years [1]. The degree to which cancer research findings are generalisable to the wider cancer population is, in part, dependent upon the representativeness of the study sample recruited [4]. One of the challenges facing population health research in cancer is the recruitment of large and representative samples of participants in a timely and cost efficient manner.

### Cancer registries as a means to access representative groups of cancer survivors

Cancer registries are a potential mechanism for recruiting population-based samples of cancer survivors for research [5]. In many developed countries cancer registries are supported by Public Health Acts or other legislation [6-8]. As a consequence, notification of any cancer diagnosis by hospitals, general practitioners (GPs), or pathology units may be legally required. Having a centralised source for recruiting a sample of cancer survivors has advantages, especially when conducting research with low incidence cancer populations such as adolescents and young adults (AYAs) [5].

Adolescents and young adults aged 14 to 19 years account for approximately 0.5% of the total population diagnosed with invasive cancers in Europe and North America [9]. The types of cancer most frequently diagnosed in AYAs differ to those diagnosed in children or older adult cancer populations. The most common cancers for AYAs include lymphoma, melanoma of the skin, and thyroid and testicular cancer[10], with these cancers also having high survival rates (5 year relative survival rate of >80%) [9]. Data from a state-based cancer registry in Australia showed that the proportion of survivors aged 14 to 19 years who were reported to be diagnosed with invasive cancers for the period 2002-2007 was slightly lower than in Europe and North America (0.4% of all new cases notified to the registry or 719 new cases in total) [11]. Of all AYA cancers diagnosed, Hodgkin lymphoma (15%), melanoma of the skin (15%), leukaemia (14%), invasive brain tumours (8%), testicular cancer (8%) and bone cancers (8%) accounted for the highest proportions [11].

For those cancer registries which are population-based, the sample of AYA cancer survivors recruited from the registry should reasonably represent the distribution of all AYA cases diagnosed. However, depending on the timing of recruitment with respect to the time which has lapsed since the individual's diagnosis, AYAs who have cancers with poor survival rates such as bone cancer and invasive brain tumours may be under-represented in research studies. Nevertheless, in the case of low incidence cancer populations such as AYAs, registries offer potential access to most cases through a single access point and may remove the need for researchers to recruit survivors from multiple sites [5].

### Process for recruitment of research participants through cancer registries

Depending on privacy legislation, there can be up to three stages of consent required when recruiting cancer survivors through registries for research studies [12]. At each stage the number of potentially eligible participants may be reduced, thereby decreasing the size and representativeness of the final sample.

In the first stage of recruitment, the registry may contact the responsible clinician and request a professional judgement as to whether the survivor is well enough for the registry to approach. Clinician consent can be active or passive, depending on the protocols in place within the registry [13,14]. Active consent requires clinicians to confirm the suitability of all identified cancer survivors prior to the registry contacting the survivor [13,14]. Passive consent requires the clinician to respond to the registry only if an identified survivor should not be contacted. If the clinician does not respond within a specified time period, clinician consent is inferred and the registry can proceed with contacting the cancer survivor [13,14].

The second stage of the recruitment process may require the cancer survivor to grant consent for the registry to provide their contact details to the researchers. Survivor consent can also be active or passive [12]. Active consent requires survivors to provide written or verbal consent if they wish to be contacted [12,14]. Passive consent allows all survivors to be contacted unless they opt-out by providing written or verbal notification that they do not wish contact to occur [12]. The registry can then provide the contact details of consenting eligible individuals to the research team [12,14]. In the third stage of recruitment the researchers contact survivors in accordance with their approved research protocol and request participant consent to take part in the research.

Recently, the authors attempted to recruit a population-based sample of AYAs with cancer to participate in a research study requiring the completion of a paper and pencil questionnaire to assess their unmet needs. Adolescents and young adults with cancer have previously been identified in the literature as a vulnerable population group reporting high levels of physical, psychological and social distress [15-18]. There is a need to understand aspects of their quality of life and unmet needs, as well as to identify risk factors for significant problems in these domains [19-21].

Recruitment was conducted through a state-based cancer registry in Australia which uses an active clinician and survivor consent process. The aim of this paper is to describe the resulting registry recruitment rates and to illustrate: 1) the impact of active clinician and survivor consent protocols on recruitment rates at stage 1, 2 and 3 of recruitment; 2) reasons for clinicians not providing consent to contact survivors; and 3) the representativeness of the final sample recruited compared with the overall AYA cancer survivor population.

## Methods

### Setting

This study was approved by the Human Research Ethics Committees of the University of Newcastle and the Cancer Institute NSW. A cross-sectional study design was used to recruit AYAs diagnosed with cancer through a state population-based cancer registry in Australia. Under the Public Health Act for the state, notification of malignant neoplasms is a statutory requirement for all public and private hospitals, departments of radiation oncology, nursing homes, pathology laboratories, out-patient departments and day procedure centres. Notifications of cancer in residents of the relevant state, who are diagnosed and treated outside of the state, are also received from other state and territory cancer registries. Demographic information and clinical details about the cancer and treating clinician are collected from notifiers [14].

### Participants

Adolescents and young adults listed on the registry were eligible to participate in the study if they were: 1) diagnosed with an invasive cancer between the ages of 14 to 19 years inclusive; 2) diagnosed between 1 January 2002 and 31 December 2007 inclusive; 3) residents of the relevant state; and confirmed by their primary treating clinician as: 4) having a life expectancy of at least 12 months; 5) physically and mentally able to complete the survey; and 6) sufficiently literate in English.

The age range used to define AYAs varies across the literature and ranges from 10 to 40 years [22-24]. In this study the upper age limit of 19 years was selected, as the World Health Organisation (WHO) defines adolescents as being between 10-19 years of age [23]. The lower limit of 14 years was chosen because, in Australia, AYAs aged 14 years and older have the legal right to make their own decisions about the type of health care they receive [25]. Survivors who did not reside in the same state as the registry could not be included as ethics approval was only granted to contact residents of that state. Adolescents and young adults diagnosed with non-invasive cancers, such as non-melanoma skin cancer (NMSC), were excluded for two reasons: 1) notification of NMSC is not legally required in most Australian states, with the exception of Tasmania (TAS), therefore records for NMSC listed on other state cancer registries are not complete or representative of the population; and 2) it was expected that the number of survivors with NMSC would be very small as most cases are diagnosed in those aged 20 years and older [11].

### Procedure

**Figure 1:**
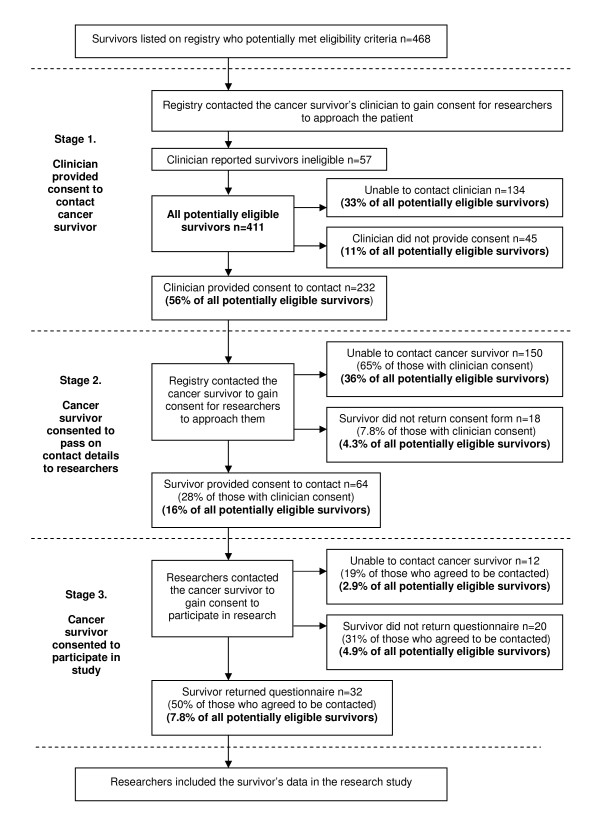
**Flowchart of the proportion of potentially eligible survivors filtered at each stage of recruitment**.

A description of the survivor recruitment protocol used in the registry is presented in Figure 1[14]. Prior to contacting clinicians, the vital status of all potentially eligible participants was confirmed by cross checking registry data with the death register for the state.

#### Stage 1- Clinicians consent to contact survivors

A letter was sent to identified AYA cancer survivors' primary treating clinicians (as recorded on the registry) to inform them about the study, confirm eligibility and request permission to contact the eligible survivor. Clinicians who did not respond to the letter within two weeks were telephoned by the registry at two-weekly intervals, up to five times, to determine the eligibility of the identified AYAs.

#### Stage 2 - Survivors consent to pass on contact details

Adolescent and young adult survivors whose clinicians consented for them to be contacted were sent a project information letter and a "consent to be contacted form" by the registry, seeking written permission to forward the survivor's contact details to the research team. Adolescents and young adults were encouraged to discuss their possible involvement in the study with their parents and/or primary treating clinicians. Survivors who had not responded within two weeks of receiving their initial letters were sent reminder letters by the registry. Survivors who had not responded within two weeks of receiving the reminder letters received follow-up telephone calls at the four-week interval. Up to two attempts to contact survivors by telephone were made. The registry provided the researchers with the contact details of AYAs who agreed to be contacted.

#### Stage 3 - Survivors consent to participate

The researchers sent AYA cancer survivors who agreed to be contacted a letter of invitation, a study information sheet and a questionnaire which asked about any unmet needs they may have experienced in the last month. Unmet needs related to: daily life; education; work; relationships; feelings; cancer treatment centre; cancer treatment staff; and information were explored. Survivors who had not returned questionnaires within two weeks of receiving them were sent reminder letters. Survivors who had not returned questionnaires within two weeks of receiving reminder letters received follow-up telephone calls at the four week interval. Up to two attempts to contact survivors by telephone were made. Return of the questionnaire was taken as implied consent to participate in the study.

### Analysis

Proportions were calculated to estimate survivor consent rates overall, and at each stage of recruitment. Fisher's exact test was performed using Stata Version 11 statistical software[26] to identify whether there were any significant differences between non-participants (AYAs who were unable to be contacted or did not provide consent) and the final sample.

Data from the registry indicated that there were 468 potentially eligible survivors. Assuming a 75% consent rate at each stage, 351 survivors would receive clinician consent at Stage 1, 263 survivors would consent to be contacted at Stage 2 and 197 survivors would consent by returning a questionnaire at Stage 3. The anticipated sample size of approximately 200 survivors would allow the estimation of consent rates with 95% confidence intervals within ±7% and a detection of differences in binary variables between the groups of 25% with 80% power at the 5% significance level.

## Results

### Recruitment rates at each stage of recruitment

The registry identified 468 cases of AYAs who met the eligibility criteria. Of these 468 potentially eligible survivors, clinicians reported that 57 did not meet the eligibility criteria. Recruitment rates for the remaining 411 survivors, including rates within each stage of recruitment, and rates for the overall sample, can be seen in Figure 1. The overall consent rate for all potentially eligible AYAs was 7.8% (n = 32).

### Reasons for clinicians not providing consent to contact survivors

Reasons reported by clinicians for not providing consent for the registry to contact survivors are presented in Table 1. Clinicians reported that 56% (n = 57) of AYAs did not meet the eligibility criteria for the study and that 35% (n = 36) were no longer their patients.

**Table 1:** Reasons provided by treating clinicians for not providing consent for the registry to contact survivors.

Reason for refusal		n = 102	%
**Survivors did not meet eligibility criteria n = 57 (56%)**			
	Not physically/mentally capable	12	12
	Diagnosis not appropriate	11	11
	<1 year life expectancy	9	8.8
	Survivor did not wish to participate	7	6.8
	Not in same state as registry	6	5.8
	Situation not appropriate	6	5.8
	Too ill	2	1.9
	Doesn't speak English	2	1.9
	Survivor unaware of cancer diagnosis	2	1.9
**Other reason n = 45 (44%)**			
	Not current patient	36	35
	No reason given	7	6.8
	Clinician did not wish to participate	2	1.9

### Comparison of the final sample compared with the overall sample

The demographic characteristics of AYAs who were included and excluded at each stage of recruitment are presented in Table 2. The final sample of participants was reasonably representative, with no significant differences between the proportions of males and females (p = 0.36) or different cancer types (p = 0.14) for those who participated and those who did not. However, there was a significant difference between the ages of the two groups, with those who participated being significantly younger than those who were unable to be contacted or did not consent (p < 0.01).

**Table 2:** Comparison of the final participant group with all other non-participants.

	Demographics of survivors							
	
	Stage 1 (n = 468)		Stage 2 (n = 232)		Stage 3 (n = 64)		Total (n = 468)	
	
	Clinician did not consent (n = 102)	Unable to contact clinician (n = 134)	Survivor did not consent (n = 18)	Unable to contact survivor (n = 150)	Survivor did not consent (n = 20)	Unable to contact survivor (n = 12)	Did not consent and unable to contact (n = 436)	Final participant group (n = 32)
	
	n (%)	n (%)	n (%)	n (%)	n (%)	n (%)	n (%)	n (%)
**Age at diagnosis**								
14-15	27 (26)	30 (22)	9 (50)	40 (27)	2 (10)	2 (17)	110 (25)*	**11 (34)**
16-17	27 (26)	51 (38)	2 (11)	46 (31)	8 (40)	6 (50)	**140 (32)**	**16 (50)**
18-19	48 (47)	53 (40)	7 (39)	64 (43)	10 (50)	4 (33)	186 (43)*	**5 (16)**
**Gender**								
Female	44 (43)	59 (44)	7 (39)	68 (45)	4 (20)	8 (67)	**190 (44)**	**17 (53)**
Male	58 (57)	75 (56)	11 (61)	82 (55)	16(80)	4 (33)	**246 (56)**	**15 (47)**
**Cancer type**								
Lymphoma	16 (16)	36 (27)	5 (28)	36 (24)	4 (20)	2 (17)	**99 (23)**	**6 (19)**
Skin	25 (25)	21 (16)	3 (17)	31 (21)	2 (10)	2 (17)	**84 (19)**	**2 (6.2)**
Testicular	9 (8.8)	13 (9.7)	0 (0)	13 (8.6)	4 (20)	0 (0)	**39 (8.9)**	**1 (3.1)**
Thyroid	11 (11)	7 (5.2)	0 (0)	11 (7.3)	2 (10)	1 (8.3)	**32 (7.3)**	**3 (9.3)**
Leukaemia	13 (13)	17 (13)	3 (17)	21 (14)	2 (10)	2 (17)	**58 (13)**	**8 (25)**
Other**	28 (27)	40 (30)	7 (39)	38 (25)	6 (30)	5 (42)	**124 (28)**	**12 (38)**

*Due to discrepancies in notified dates of diagnosis one non-participant may have been under 14 years of age and six non-participants may have been over 19 years of age at diagnosis.

**"Other" includes cancers of brain, bone, connective tissue, colorectum, small intestine, kidney, liver, ovary, bladder, breast, lung and heart.

## Discussion

### Summary of results

Despite the potential of the registry to access a large, population-based sample of AYAs diagnosed with cancer, only a small percentage (7.8%) of the potentially eligible population was recruited into the study. Compared with AYAs who were unable to be contacted or did not provide consent, the resulting sample did not differ significantly by gender or cancer type. However, compared with all potentially eligible AYAs listed on the registry within the time period of interest, a greater proportion of those recruited into the study sample were 14 to17 years of age. Given the potential promise of registry recruitment to deliver population-based, representative samples, it is important to understand what factors may have contributed to these low recruitment rates and potential sample bias.

### Potential reasons for low recruitment rates and non-representative samples

### Clinician-related reasons

#### Clinicians no longer had contact with survivors

In the current study, over one-fifth of potential participants could not be contacted by the registry because their clinicians refused to provide consent. Surprisingly, survivor ineligibility was only cited as a reason for 56% of these cases. For the majority of the remaining survivors for whom clinician consent was not obtained, it was because they were no longer current patients of the clinician (35%). This situation may arise because registries receive cancer notifications from various sources including pathology laboratories, survivors' GPs, or cancer specialists [27,28]. Depending on the source of the notification, the provider who is listed as the treating clinician on the registry file may not be the person who is overseeing the survivor's cancer care. Factors such as increasing population mobility[29,30] and an emphasis on consumer satisfaction in health care[31] may also mean that people are likely to change GPs as their needs change. This may contribute to the lack of continuity in the doctor-patient relationship.

#### Clinicians did not respond

Almost a third (n = 134, 33%) of all identified potential participants could not be contacted because their clinicians did not respond to correspondence from the registry. Reasons for not responding can only be hypothesised. However, one explanation may be that, as outlined previously, the identified survivor was no longer a current patient of the treating clinician. Further, clinicians are very time poor [32,33]. This factor may be of particular importance when active clinician consent is required by the registry. Others may feel that responding to or participating in research studies is not an important part of their professional role [34,35]. Members of particular professional groups or clinical sub-specialities may have different attitudes towards research participation, and this may in turn influence the types of survivors who are permitted to be contacted and who subsequently participate in the study [32,36].

### Survivor-related reasons

#### Participants were unable to be contacted

Almost two-thirds (65%) of the 232 AYAs who received their clinician's consent to be approached by the registry could not be contacted due to changed contact details. Although this may present a problem for all retrospective studies recruiting through cancer registries, it may be a particular problem when recruiting AYAs, as individuals in this age group are highly mobile [30,38]. In Australia, half of all young people aged 15-24 years moved residence during 1997 to 2001, with a large proportion moving interstate [30]. This problem may not be so prevalent for older survivor groups [39]. Australian registries can cross-check adult survivors' names and dates of birth with the electoral role and check for changes of address. However, the same procedure cannot be used to update contact information for AYAs under the age of 18 years who are ineligible to vote.

#### Participants did not want to be contacted by the researchers

It might be expected that the low participation rate achieved was the result of lack of interest in the research among AYAs. However, only a small number of survivors refused to be contacted about the research (n = 18, 7.8%). This suggests that being contacted for participation in research studies is acceptable to this survivor group.

### Implication of low recruitment rates and non-representative samples

There are ethical, research and cost related implications of low recruitment rates and non-representative samples achieved through registries using active clinician consent.

#### Ethical implications

The process of seeking active clinician consent is underpinned by the ethical principle of beneficence. It is implemented to minimise avoidable psychological harm that survivors might experience by being contacted by the registry [12,40]. However, in the current study the main reasons for potential participants being excluded at stage 1 of recruitment were related to clinicians no longer having professional relationships with survivors, or clinicians simply not responding. Forty-three percent of all identified AYAs could not be approached due to clinician refusal or non-response. This suggests that a large proportion of survivors are being excluded for reasons not related to study eligibility or emotional health, indicating that the balance between the ethical principles of beneficence and patient autonomy may need to be considered [12,41,42].

In weighing up these two ethical principles, the potential level of harm associated with the research and its probability of occurrence need to be explored. It is generally accepted that most research studies will involve some potential for harm [40,42]. Harm may range from simple inconvenience to psychological distress, or in the case of drug trials, unforseen side effects. Prior to commencing a study, researchers need to provide a justification to relevant ethics committees as to why the potential benefits of their research outweigh any potential risks [40,42]. In many studies, risk of harm may be small compared to the potential benefits of the research [43-45].

#### Research implications

Participation in research may have benefits for both the individual and the community. Patients who participate in research studies may report better outcomes than those who do not participate [46,47]. Survivors have also reported valuing altruistic benefits of participation [48]. Research may lead to improved outcomes for the wider cancer population [49-52]. Therefore, it is necessary to weigh up the risk of harm to individuals with the potential research benefit to the individual and to the wider cancer population [44,45,49].

If neither a high consent rate nor representative sample is obtained, the results of a study may not be generalisable to the wider cancer population [53]. In this study, the needs reported by the 32 AYAs who completed the questionnaire may not represent the needs of the AYA population. The small sample size also meant that there was limited statistical power to test hypotheses or to validate the instrument developed for the study.

Recruiting through more than one registry may be one way to increase the sample size. However, if low consent rates are achieved in all registries, the potential for bias in the sample remains. Other issues including the burden of gaining approval from numerous cancer registry ethics committees, as well as the cost of research and registry personnel, would also need to be taken into account [54].

#### Cost-related implications

There are high public and individual costs involved in conducting research. The vast majority of research is publicly funded through either large government organisations such as the National Cancer Institute (NCI) in the United States and the National Health and Medical Research Council (NHMRC) in Australia, or through charitable organisations [55,56]. For this reason, it is of paramount importance that the public gets a good return on investment. However, the process of recruitment through registries, especially when active clinician consent is required, can be expensive in terms of time, energy and resources[54], and for some population groups may not be cost-efficient. In the present case where only 32 AYAs were recruited from 411 potentially eligible AYAs, the enormous staff and material costs of the research appear to be out of proportion to the number of participants recruited and usefulness of the of data collected.

There are also costs to individual clinicians and survivors who participate. The active consent protocol adds an additional burden to already time-poor clinicians [54]. This would appear to be an inefficient use of time for both the registry and the clinician, particularly if clinicians no longer have contact with these survivors [12].

Furthermore, the active consent protocol places a burden on survivors. In the current study, there is a cost to the 32 AYAs who participated. Despite the time and effort expended by participants, it is difficult to do anything meaningful with their data due to the low overall response rate. In light of the cost and limited usefulness of the data obtained, it may be important to explore possible alternatives that could be used to overcome these low consent rates.

### Resource efficient alternatives for achieving representative research samples

There are a number of alternative protocols which could be considered as feasible options when attempting to recruit population-based samples through cancer registries.

#### Passive clinician consent

The registry involved in the current study required the use of an active clinician consent protocol, however, some registries allow passive clinician consent [12,13]. Passive clinician consent may help to reduce the burden placed on clinicians and has been used successfully by a number of international registries and studies [12,13]. However, given the fact that many clinicians may no longer have contact with AYA survivors, this method may still not be viable.

Other approaches could involve the registry giving clinician and survivor details directly to researchers who then make the initial contact with the clinician, instead of the registry. Over 60% of cancer registries in the United States use this approach [12]. The majority of registries using this researcher-initiated protocol require passive clinician consent (70% of registries) and a survivor opt-out approach (86% of registries) [12]. However, in Australia, because registries are usually notified under a Public Health Act, information cannot be provided to a third party without survivor consent. Furthermore, the process of researchers notifying a survivor's clinician prior to contacting the survivor about a research study may still be viewed as paternalistic. Beskow and colleagues reported that over two-thirds of patients (68%) said that they preferred that researchers contact them directly about opportunities for research participation, rather than checking with their physicians first [57]. Therefore, alternatives to a passive clinician consent model should be explored.

#### Direct survivor consent

A novel method for assisting registries to provide cancer survivors with information about research studies for which they may be eligible could be through direct survivor contact [57,58]. This might involve a one-off contact with a survivor by the registry which would allow the survivor to indicate whether or not they would like to be contacted about research studies in the future, the types of research they would like to be contacted about, and how often these contacts could be made [5]. This consumer driven approach would allow survivors to choose the focus of research studies they are interested in participating in and negate the need for clinician consent to contact the survivors.

A national household survey in Britain found that although 82% of participants did not know the registry existed, 95% thought that the information collected was useful [58]. Over 80% of respondents indicated that the collection personal information by the registry and the use of the registry for recruitment purposes was acceptable. In anticipation of low survivor awareness of the registry and its role, cancer registries could send an information leaflet as part of their initial contact with survivors.

#### Recruitment of AYAs via treatment centres and clinics

Direct consumer contact may provide greater survivor autonomy and overcome low clinician response rates. However, this method may not overcome difficulties associated with obtaining up-to-date contact details of survivors. Recruitment of AYAs from treatment centres and clinics may potentially address this problem. Due to the low prevalence of cancer in AYAs, recruitment from a number of different clinics is likely to be required in order to achieve a sufficiently large and representative sample. A number of AYA research studies have successfully used this method of recruitment [59,60]. One complication may be the need to target both adult and children's hospitals to obtain a sample which adequately covers the age range of the AYA population.

### Limitations

Limitations should be considered when interpreting the findings of the current study. First, recruitment was only conducted through one state registry in Australia. Therefore, it is possible that the low recruitment rates obtained may have been specific to this registry. However, studies which have compared recruitment rates between registries using active or passive clinician consent have generally found that recruitment rates are lower in registries requiring active consent [13]. Second, 62% of AYAs who consented and could be contacted returned a completed survey. It is possible that telephone administered or online surveys might have been more acceptable to this group than the paper and pencil format that was used. However, some studies using online methods of data collection with this population have also resulted in small sample sizes [61].

## Conclusions

Despite the potential for cancer registries to provide researchers with access to large and representative samples, current registry protocols such as active clinician consent may inhibit this process. Alternative methods such as passive clinician consent or direct survivor consent may help to overcome some of these barriers. In the case of AYAs diagnosed with cancer who are highly mobile, recruitment through registries may not be feasible and other alternatives, such as recruitment through treatment centres and clinics, may need to be considered.

## Competing interests

The authors declare that they have no competing interests.

## Authors' contributions

RSF, TCM, and MC were the initiators of the manuscript. TCM and RSF contributed to the study design, coordinated participant recruitment, and managed data collection. ET provided expert advice regarding registry protocols. MC and TCM undertook the statistical analysis. ET assisted with the interpretation of data. All authors contributed to drafting, revising and approving the final manuscript.

## Pre-publication history

The pre-publication history for this paper can be accessed here:

http://www.biomedcentral.com/1471-2288/11/5/prepub
